# Comparative studies on mannan and imiquimod induced experimental plaque psoriasis inflammation in inbred mice

**DOI:** 10.1093/cei/uxad004

**Published:** 2023-01-16

**Authors:** Huimei Wu, Jiaxin Ou, Kangxin Li, Tingting Wang, Kutty Selva Nandakumar

**Affiliations:** Southern Medical University—Karolinska Institute United Medical Inflammation Center, School of Pharmaceutical Sciences, Southern Medical University, Guangzhou, China; School of medicine, Southern University of Science and Technology, Shenzhen, China; Southern Medical University—Karolinska Institute United Medical Inflammation Center, School of Pharmaceutical Sciences, Southern Medical University, Guangzhou, China; Guangdong Provincial Key Laboratory of New Drug Screening and Guangzhou Key Laboratory of Drug Research for Emerging Virus Prevention and Treatment, School of Pharmaceutical Sciences, Southern Medical University, Guangzhou, China; Southern Medical University—Karolinska Institute United Medical Inflammation Center, School of Pharmaceutical Sciences, Southern Medical University, Guangzhou, China; Department of Endocrinology, Fifth Affiliated Hospital, Southern Medical University, Guangzhou, China; Southern Medical University—Karolinska Institute United Medical Inflammation Center, School of Pharmaceutical Sciences, Southern Medical University, Guangzhou, China; State Key Laboratory of Oncology in South China, Collaborative Innovation Center for Cancer Medicine, Sun Yat-sen University Cancer Center, Guangzhou, Guangdong, China; Southern Medical University—Karolinska Institute United Medical Inflammation Center, School of Pharmaceutical Sciences, Southern Medical University, Guangzhou, China; Department of Environmental and Biosciences, School of Business, Innovation and Sustainability (FIH), Halmstad University, Halmstad, Sweden

**Keywords:** psoriasis, innate immune cells, γδ T cells, IL-23/IL-17 axis, mannan, IMQ

## Abstract

Psoriasis is a genetically determined, environmentally triggered, immune system-mediated autoimmune disease. Different animal models are needed to investigate the complex pathological mechanisms underlying this disease. Therefore, we established mannan-induced psoriasis model and compared with the most commonly used imiquimod-induced psoriasis in terms of disease, induction of innate immune cells, expression of cytokines, and the effect of dexamethasone treatment. Mannan significantly induced more severe psoriasis with better disease relapsing feature than imiquimod (IMQ). As determined by immunohistochemistry, IMQ induced significantly more infiltration of CD11c^+^ and F4/80^+^ cells than mannan in the skin. However, cytometric analysis showed a significant increase in the percentage of Gr-1^+^ neutrophils in the spleen and lymph nodes as well as F4/80^+^ macrophages in the spleen after mannan exposure. Variation in the percentage of significantly increased Vγ4 T cells was also found to be dependent on the lymphoid organs tested. However, there is a clear difference between these models in terms of expression of certain cytokine genes: IL-22, IL-23, IL-17E, and IL-17F were expressed more predominantly in mannan-induced inflammation, while IL-6 and IL-17A expressions were significantly higher in IMQ model. Interestingly, dexamethasone treatment strongly reduced epidermal thickness and histological scores induced by mannan than IMQ. Despite inducing psoriasis-like inflammation, certain differences and similarities were observed in the immune responses induced by mannan and IMQ. However, mannan-induced psoriasis model is relatively more simple, economical and less harmful to mice with an increased possibility to develop a chronic psoriasis model by exposing mice to mannan.

## Teaser

Epicutaneous application of mannan mixed with incomplete Freund’s adjuvant induced clinical symptoms of plaque psoriasis in inbred mice. Although certain similarities and differences exist in the involvement of neutrophils, macrophages, dendritic cells, Vγ4 T cells, and pro-inflammatory cytokines between mannan- and imiquimod-induced psoriasis-like inflammation, former model was more severe with a better relapsing disease feature and treatment response. In addition, mannan-induced psoriasis is relatively easy to induce, economical and less harmful to mice with an increased possibility to develop a chronic psoriasis model in mice with repeated exposures.

## Graphical Abstract

**Graphical Abstract:**
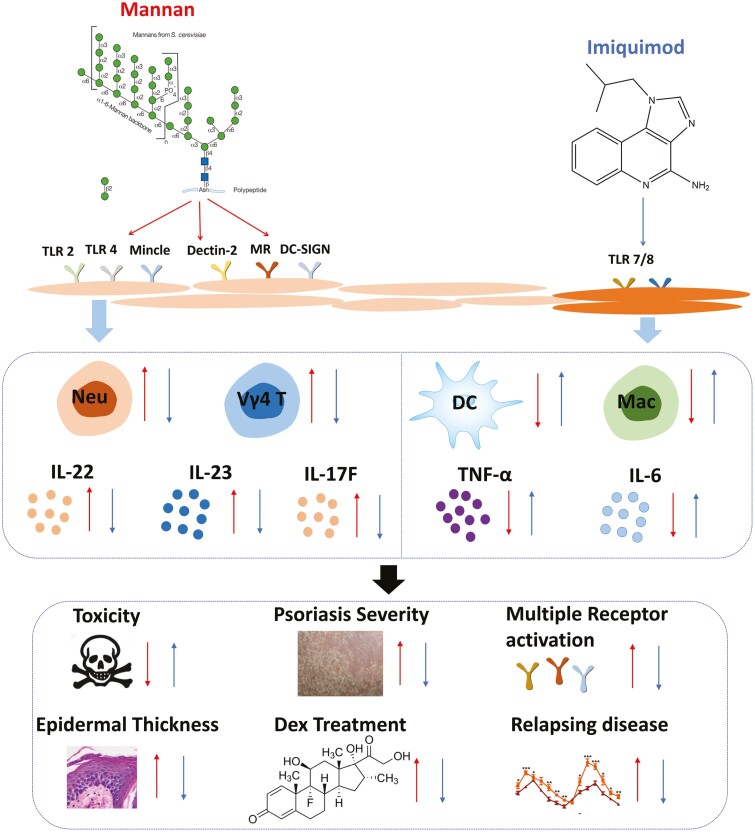

## Introduction

Psoriasis is an inflammatory skin disorder, which affects 25 million people in North America and Europe [1]. Although etiology of psoriasis is still not clear, a strong genetic association to major histocompatibility complex and several environmental factors were reported [2]. Psoriasis skin is often characterized by hyperproliferation of keratinocytes in epidermis, increased angiogenesis and immune cell infiltrations in the dermis. Both innate and adaptive immunity are crucial for the disease pathogenesis [3]. Innate immune cells, such as macrophages, neutrophils, and dendritic cells activated by TNF-α and IL-23, modulate functions of adaptive effector cells like Th17 cells in psoriasis secreting IL-17 family of cytokines [4], which in turn induce the activation of keratinocytes and the production of anti-microbial peptides, CXCL10, CCL5, CXCL8, and TNF-α [5]. IL-23/IL-17A inflammatory axis is critical for the initiation and amplification of inflammatory responses in psoriasis [6]. IL-23 can strongly promote γδ T cells to produce IL-17A in γδ T cell-mediated skin diseases [7] and several γδ T-cell subsets with distinctive functions exist in the skin tissue: dendritic epidermal T cells, which uniformly express an invariant Vγ5Vδ1 TCR exclusively reside in the murine epidermis and Vγ4 T cells, a dominant subset of murine peripheral γδ T cells, have also found to be distributed in the dermal layer of the murine skin [8, 9]. Importantly, Vγ4 T cells were identified as a major source of IL-17A, a cytokine of major importance in the skin diseases [10]. The immune cells identified to be crucial in the induction of psoriasis-like skin inflammation in the mouse strains are also essential in human plaque psoriasis.

Various psoriasis models (spontaneous, genetically engineered, human skin transplant, and induced mouse models) are being used for exploring the mechanisms underlying psoriasis development and for testing potential new drugs. However, each model has its own advantages and disadvantages [11]. Among these psoriasis models, most commonly used and classical one is imiquimod-induced psoriasis. Epicutaneous application of imiquimod (IMQ) for 5–7 days leads to the development of an acute psoriasis phenotype, but it is often accompanied with severe weight loss [12]. IMQ is a selective synthetic agonist to toll-like receptor 7/8 (TLR7/8) [13], though it is advantageous, this specificity precludes mimicking many natural ligands that are involved in the induction of psoriasis. On the other hand, an intraperitoneal injection of mannan extracted from *Saccharomyces cerevisiae* cell wall was shown to induce both psoriasis and psoriatic arthritis symptoms in reactive oxygen species (ROS) deficient Ncf1 gene-mutated mice that are available only in certain labs [14]. Therefore, we developed a variation of mannan-induced psoriasis model by epicutaneous application in inbred mice, a natural route of antigen exposure in the skin, which showed a better disease relapsing feature. We compared this model with IMQ-induced psoriasis-like skin inflammation.

## Materials and methods

### Mice

Eight to twelve weeks old BALB/c, C57BL/6J, C57BL/6NQ, and KM female mice maintained in a pathogen-free animal house were purchased from Southern Medical University and Guangdong Medical Animal Experiment Center. All animal experiments were performed in accordance with the guidelines of the National Institutes of Health (NIH Publication No. 8023) and approved by the ethics committee of Southern Medical University (l2018183). Mice were placed in cages, in a climate-controlled environment having 12-h light/dark cycles. All the procedures were approved and supervised by Southern Medical University Animal Care and Use Committee, Guangzhou, China.

### Mannan or IMQ induced psoriatic skin inflammation and dexamethasone treatment

In all the mice used in psoriasis experiments, back skin with an area of 2.0 × 3.5 cm was shaved after anesthetized with isoflurane (Rwd, Guangdong, China). At first, we titrated the concentration of mannan with 100 μl of 30, 50, 80, or 100 mg/ml mannan solution (Sigma-Aldrich, Missouri, USA). Second, 100 μl of mannan at 50 mg/ml was mixed with 100 μl of incomplete Freund’s adjuvant (IFA, Sigma-Aldrich), complete Freund’s adjuvant (CFA, Sigma-Aldrich), paraffin oil (Aladdin, Shanghai, China), or 100 g of Vaseline (Aladdin) to test optimal formulation. Third, we used 5 mg of mannan mixed with IFA to select the optimal mouse strains by testing this model in BALB/c, C57BL/6J, C57BL/6NQ, and KM mice. Mannan was applied continuously for 3 days in each experiment. Erythema, scales and skin thickness were monitored daily for at least 9 days. Five mg of mannan mixed with IFA in a ratio of 1:1 for three consecutive days on the back of BALB/c mice was used in all the follow-up experiments. To induce a relapsing disease, mannan was topically applied again for 3 days with the same concentration for secondary stimulation after symptoms of psoriasis disappeared on day 9. Fifty mg of IMQ cream (5% IMQ, MedShine, Sichun, China) was applied on the back of mice for five consecutive days for one time stimulation. Mice recovered from symptoms around day 10 were used for second time stimulation with IMQ similarly for 5 days by topical application. Psoriasis area and severity index (PASI) was used to evaluate the disease symptoms, which contain erythema, scales and thickness. Each parameter received four points with a total points of twelve. 0, no symptoms; 1, mild; 2, moderate; 3, severe; 4, very severe. Digital caliper (Neill-Lavielle, Kentucky, USA) was used to measure the skin thickness. We recorded PASI at the same time during each day. Eight mg/kg dexamethasone was injected subcutaneously from days 0–4 to treat the skin inflammation in both the experimental models. In the treatment experiment, skin samples were harvested at day 4 and used for H&E staining.

### Histology

Skin samples from each group were harvested at the peak of psoriasis (day 4), and fixed in 4% paraformaldehyde for 24 h at room temperature (RT). These samples were dehydrated and treated with different concentrations of alcohol and dimethylbenzene. Skin samples were embedded in liquid paraffin at 70°C using a paraffin embedding machine (Leica, Solmas, Germany) and cut into 8 μm sections using a Paraffin microtome (Leica). The sections were de-paraffinized and dehydrated firstly, before staining with hematoxylin solution for 3 min and then 0.2% eosin (Beyotime, Shanghai, China) for 5 min. Images were acquired using an upright optical microscope camera (Nikon Ci-E, Tokyo, Japan) with 10× magnification. To measure the thickness of epidermis, six random regions from each mouse skin were chosen. Baker’s scores [15] were used to evaluate the pathological condition of the affected skin from each group.

### Immunohistochemistry

Frozen slices were prepared for immunohistochemistry by dehydrating in 30% sucrose-PBS solution for 48 h at 4°C. Skin samples were embedded in optimal cutting temperature compound (OCT, Sakura Finetek, California, USA) and frozen quickly overnight at −80°C. Eight μm sections were cut by using a microtome (Leica) and stored at −20°C until used. Before staining with antibodies, 5% goat serum was used for blocking, and then tissues were dehydrated with 75–95% ethanol. Innate immune cells were stained with biotin-rat anti-mouse Gr-1 (1:200, BioLegend, California, USA), CD11c (1:100, BioLegend), or F4/80 (1:100, BioLegend) antibodies for 1 h, followed by an incubation with streptavidin-HRP (1:800, Yeasen, Shanghai, China) for 40 min. DAB solution (Vector Laboratories, California, USA) was used for color development and hematoxylin (Phygene, Shanghai, China) was used for counterstaining before visualization under the microscope (Leica). F4/80 and Ly6G positive cells in each mm^2^ were calculated under an optical microscope.

### Immunofluorescence

Skin tissues were harvested at the peak of psoriasis, cut into 6-μm-thick sections, frozen until used, and then stained using a standard procedure. Biotin-rat anti-mouse CD11c (1:100, Biolegend) were used as primary antibodies and incubated overnight at 4°C, whereas the secondary reagent was Alexa Fluor® 488 conjugated streptavidin (1:800, Biyotimes, Shanghai, CA). After PBST (0.2% Tween-20 in PBS) washing, the tissue slices were fixed with Vectashield® containing DAPI and used for visualization. Frozen tissue sections were examined using a confocal microscope (Nikon, Tokyo, Japan), and images were captured at 20× magnification. CD11c positive cells in each mm^2^ were calculated under a confocal microscope.

### Flow cytometry

Single cells were harvested using 45 μm filters (Biologix, Shanghai, China) from spleen and draining lymph nodes at the peak of psoriasis. At first, cells were washed three times with PBS. Surface staining of innate immune cells was performed with fluorescent-labeled antibodies: F4/80-PerCP-Cy 5.5, CD11c-PE, CD11b-APC, and GR-1-FITC. Staining of γδ T cells and their subsets was performed with CD3-APC, γδ TCR-PE, TCR-β-BV421, Vγ4-FITC, and Vγ5-BV510 (BD Biosciences, New Jersey, USA) antibodies for 30 min at RT. FACS was done using LSR II (BD Biosciences) and data were analyzed using Flow Jo version 7.0 (Tree Star, California, USA).

### RNA isolation and RT-PCR

Total RNA was extracted from the skin with Trizol reagent (Invitrogen, California, USA) and dissolved in RNAse-free DEPC water (Phygene, Shanghai, China) before analysis. Total RNA was reverse transcribed to cDNA by using a reverse transcription Kit with gDNA Eraser (Thermo Scientific, Massachusetts, USA) following manufacturer’s instructions. Each RT-PCR reaction was performed with SYBR Premix Ex Taq II Tli RNaseH Plus (Takara biotech, Osaka, Japan) and a LightCycler 96 was used for detection (Roche, Basel, Switzerland). For quantitative control, β-actin was used. Transcript levels were calculated relative to the control and the relative fold inductions were calculated using the 2^−ΔΔCt^ algorithm. Specific primers used for detection are shown in Table 1.

**Table 1.** List of primer sequences

Target gene	Direction	Primer sequence (5 to 3)
β-actin	Forward	ACCGTGAAAAGATGACCCAG
	Reverse	GTACGACCAGAGGCATACAG
TNF-α	Forward	ACGCTCTTCTGTCTACTGAACT
	Reverse	ATCTGAGTGTGAGGGTCTGG
IL-6	Forward	GAGAAAAGAGTTGTGCAATGGC
	Reverse	CCAGTTTGGTAGCATCCATCAT
IL-17A	Forward	CCCCTAAGAAACCCCCACG
	Reverse	TAAAGTCCACAGAAAAACAAACACG
IL-17E	Forward	ACAGGGACTTGAATCGGGTC
	Reverse	TGGTAAAGTGGGACGGAGTTG
IL-17F	Forward	GTCAGGAAGACAGCACCA
	Reverse	AGCCAACTTTTAGGAGCA
IL-22	Forward	CATGCAGGAGGTGGTACCTT
	Reverse	CAGACGCAAGCATTTCTCAG
IL-23-P19	Forward	AGCAACTTCACACCTCCCTAC
	Reverse	ACTGCTGACTAGAACTCAGGC
CD206	Forward	TCCGTCACCCTGTATGCCT
	Reverse	TCCACAATCCCGAACCTTT
TLR4	Forward	AGAGCCGTTGGTGTATCTTTG
	Reverse	CCCATTCCAGGTAGGTGTTTC
DC-SIGN	Forward	CTGGCGTAGATCGACTGTGC
	Reverse	AGACTCCTTGCTCATGTCAATG
TLR2	Forward	CTCTTCAGCAAACGCTGTTCT
	Reverse	GGCGTCTCCCTCTATTGTATTG
Dectin-2	Forward	TTCTTACTTCCTGGGTCTTTCG
	Reverse	AACACACCGCTCTTCTGGA
Mincle	Forward	TGTCGTAACATATCGCAGCTC
	Reverse	GGACAGCAATTCTTGACTGAACC

### Statistical analysis

The data were analyzed with GraphPad Prism 5 and are presented as mean ± SEM. Two-tailed unpaired Student’s *t* test was used for comparison between two groups. One-way analysis of variance with Bonferroni or Newman–Keuls correction was used for multiple comparisons. Probability values <0.05 were considered as significant for 95% confidence interval.

## Results

### Mannan-induced psoriasis-like skin inflammation in inbred mouse strains

**Figure 1:**
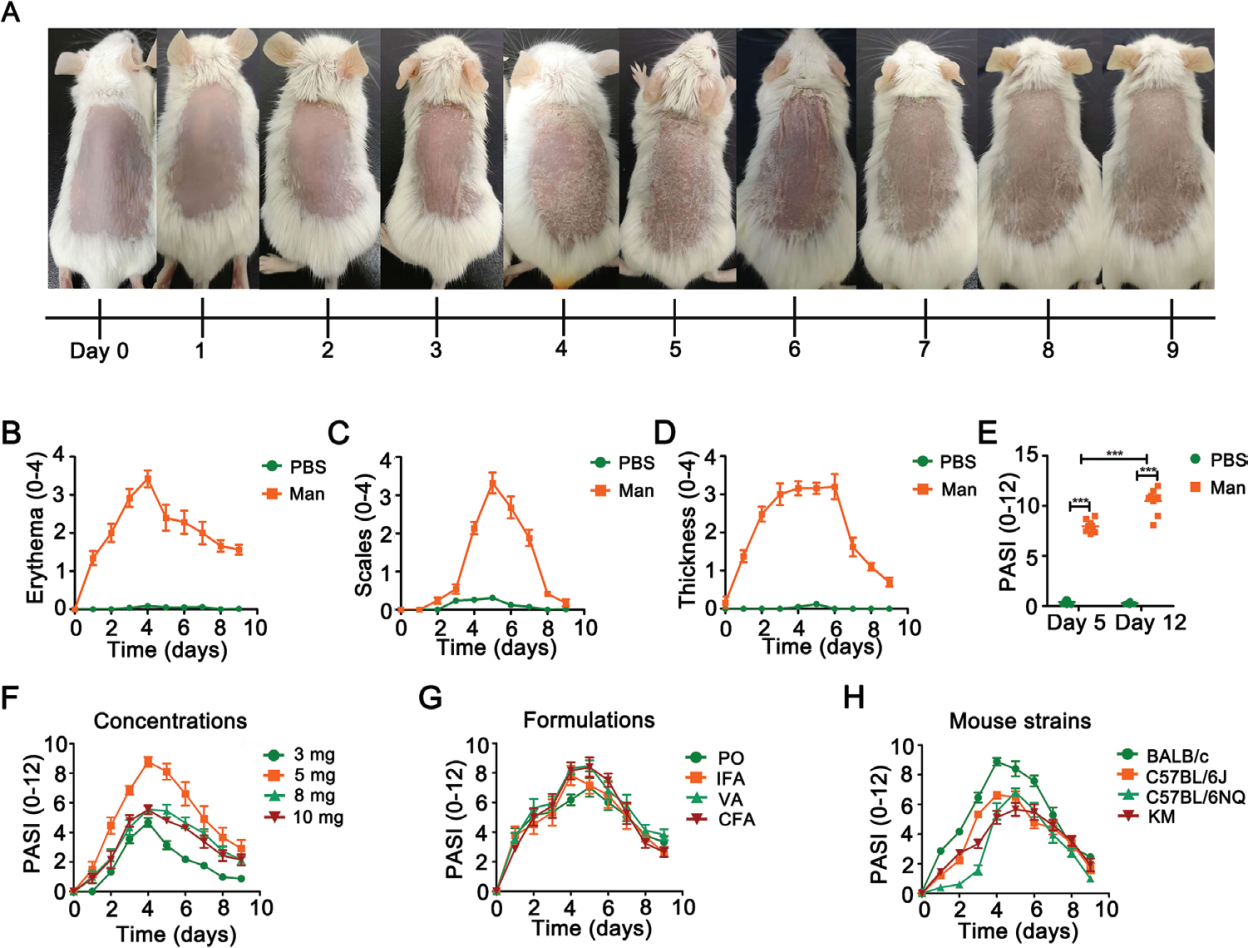
Development and characterization of MISI. (**A**) Clinical features of mannan-induced psoriasis from days 1 to 9 (*n* = 10/group). (**B**) Erythema, (**C**) scales and (**D**) skin thickness after mannan or PBS application in BALB/c female mice on three consecutive days starting from day 0 (*n* = 5/group). (**E**) Total PASI at days 4 and 12 after one or two times mannan applications (*n* = 8/group). (**F**) 3, 5, 8, or 10 mg of mannan was tested for inducing psoriasis (*n* = 10/group). (**G** and **H**) Formulation and mouse strains for an optimal induction of the disease (*n* = 10/group). Each experiment was repeated two times. Man, mannan; PO, paraffin oil; IFA, incomplete Freund’s adjuvant; VA, Vaseline; CFA, complete Freund’s adjuvant. Statistical analyses were performed using an unpaired *t* test and *n*, indicates number of mice used in each group. The data represent mean ± SEM. ***P < 0.001.

We established a new psoriasis model in BALB/c mice with mannan extracted from *S. cerevisiae* cell wall. Psoriasis induced by mannan and IFA mixture lasted for 9 days. Erythema started at day 3 with white scales, while epidermal thickness increased from day 4 onwards (Figure 1A). We found clear erythema, scales and an increase in the skin thickness after three consecutive days after mannan application, which reached to the peak level around day 4 or 5 (Figure 1B-D). The peak of psoriasis disease at day 5 had a mean maximum PASI of 7.9 ± 1.0, while the relapsing disease peak had severe symptoms and higher PASI of 10.0 ± 2.0 at day 12 after repeated exposures to mannan (Figure 1E). Different concentrations (3, 5, 8, and 10 mg) and formulations of mannan in IFA, CFA, Vaseline, or paraffin oil were used to find the optimal conditions to induce psoriasis inflammation. In addition, various mouse strains (BALB/c, C57BL/6NJ, C57BL/6NQ, and KM) were tested for psoriasis susceptibility. Our results demonstrate that 5 mg of mannan (Figure 1F) mixed with incomplete Freund’s adjuvant (Figure 1G) in BALB/c mice (Figure 1H) was optimal for inducing psoriasis-like skin inflammation, though we did not find any significant differences in the disease induction between different formulations. To have an easy preparation protocol, IFA was chosen.

### Comparison of skin inflammation induced with mannan and IMQ

**Figure 2:**
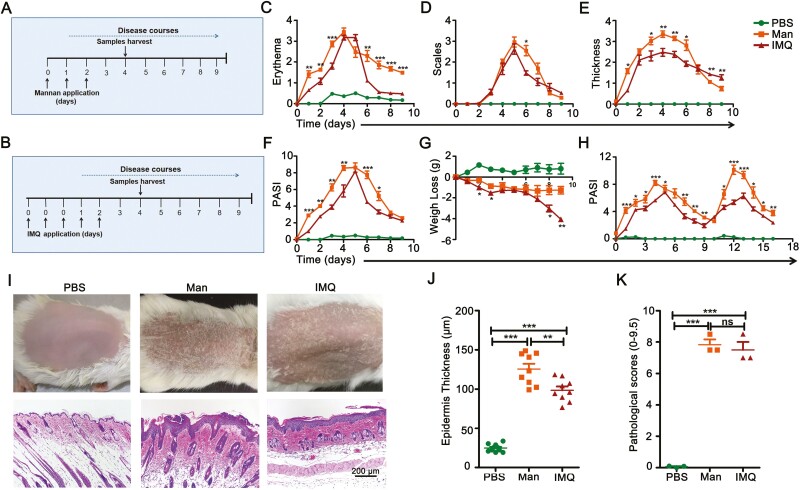
Comparison between mannan and imiquimod-induced skin inflammation. (**A**) Schematic diagram for topical application of mannan and IFA mixture or (**B**) IMQ on BALB/c mice for disease development. (**C–F**) Comparison of erythema, scales, skin thickness as well as total PASI in BALB/c mice after PBS, mannan or IMQ treatment (*n* = 10/group). In order to show the degree of erythema, scales and skin thickness between two models for comparison, we considered the application of IMQ for the first three days as days −2, −1, and 0. (**G**) Comparison of weight loss (*n* = 10/group), (**H**) relapsing disease (*n* = 10/group), (**I**) clinical and histological features at day 4 (*n* = 5/group), and (**J** and **K**) epidermal thickness and pathological scores (*n* = 3/group) with mannan and IMQ. Scale bars: 200 μm. Each experiment was repeated two times. Man, mannan; IMQ, imiquimod. Statistical analyses were performed using an unpaired *t* test and *n*, indicates number of mice used in each group. The data represent mean ± SEM. ns, not significant. *P < 0.05; **P < 0.01; ***P < 0.001.

We compared mannan-induced psoriasis mouse model with the commonly used IMQ psoriasis model. Application methods, psoriasis scores, clinical symptoms, and histological scores were compared. To induce psoriasis-like inflammation, 5 mg of mannan mixed with IFA in a ratio of 1:1 was applied for three consecutive days (Figure 2A), whereas, 50 mg of Aldara (5% IMQ) cream was applied on the back of the mice for five consecutive days (Figure 2B). Results show that mannan-induced skin inflammation (MISI) has higher scores of erythema than IMQ-induced psoriasis (IISI) on several days (Figure 2C), while differences in the scales were observed only at day 6 between these two groups (Figure 2D). Skin thickness and total PASI were higher in MISI than IISI (Figure 2E-F), however, later group of mice applied with IMQ had severe weight loss (Figure 2G). During disease relapse, psoriasis symptoms were significantly more severe with mannan than IMQ (Figure 2H). Clinical symptoms and histological staining are shown in figure 2I. A significant level of keratinocyte proliferation was observed after mannan application. In comparative terms, mice developed a higher level of epidermal thickness after mannan application compared to IMQ, while no significant differences in the histological scores between these two groups were observed (Figure 2J-K).

### Involvement of inflammatory cells in the psoriasis mouse model

**Figure 3:**
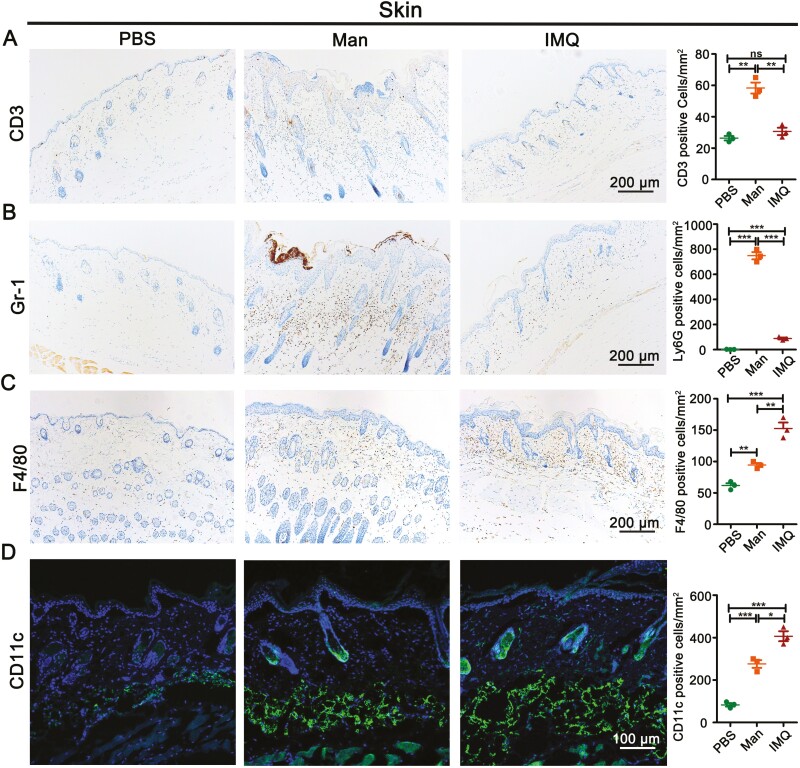
Expression of inflammatory cells in the skin after mannan or IMQ application. Representative pictures of immunohistochemical and immunofluorescence staining showing (**A**) CD3^+^_,_ (**B**) Gr-1^+^_,_ (**C**) F4/80^+^, and (**D**) CD11c^+^ cells in the psoriasis skin after mannan or IMQ application at day 5 (*n* = 3/group). (A–C), scale bar = 200 μm; (D), scale bar = 100 μm. Man, mannan; IMQ, imiquimod. Statistical analyses were performed using an unpaired *t* test and *n*, indicates number of mice used in each group. The data represent mean ± SEM. ns, not significant; *P < 0.05; **P < 0.01; ***P < 0.001.

**Figure 4:**
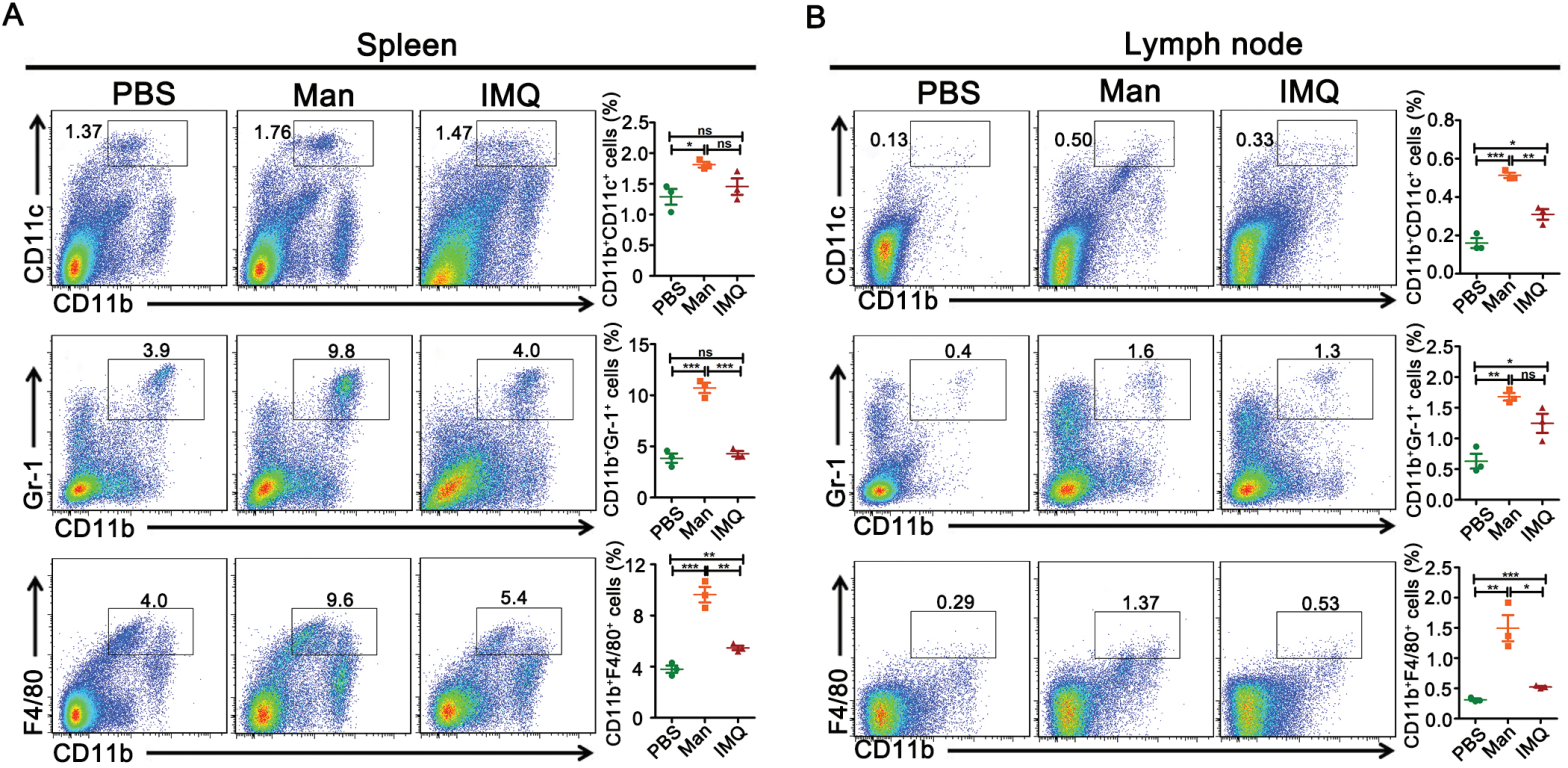
Percentage of innate immune cells in the spleen and lymph nodes after mannan or IMQ exposure. FACS results showing percentage of dendritic cells (CD11b^+^CD11c^+^), neutrophils (CD11b^+^Gr-1^+^), and macrophages (CD11b^+^F4/80^+^) present during MISI and IISI in (**A**) spleen and (**B**) draining lymph nodes (*n* = 3/group). Man, mannan; IMQ, imiquimod. Statistical analyses were performed using an unpaired *t* test and *n*, indicates number of mice used in each group. The data represent mean ± SEM. ns, not significant; *P < 0.05; **P < 0.01; ***P < 0.001.

Dysregulated interactions of innate and adaptive immune cells contribute to the development of psoriasis. Although expression of few T lymphocytes in these two psoriasis models was observed, a higher level of CD3^+^ T-cell expression was found in MISI (Figure 3A). Since human psoriatic lesions contain innate immune effector cells, such as neutrophils, CD11c^+^ DCs, and macrophages [1], we detected the level of neutrophils, CD11c^+^ DCs, and macrophages in the skin, spleen, and draining lymph nodes from these two experimental psoriasis models. Immunohistochemistry and immunofluorescence analysis of skin samples revealed a significantly higher level expression of CD11c^+^ DCs and F4/80^+^ macrophages but not Gr-1^+^ neutrophils in IMQ than mannan applied mice (Figure 3B-D). On the other hand, flow cytometric analysis showed a significantly increased percentage of CD11b^+^Gr-1^+^ neutrophils in the spleen as well as CD11b^+^F4/80^+^ macrophages in the spleen and lymph nodes of mannan applied group (Figure 4A-B). These results indicate a variation in the contribution of different innate immune cells from secondary lymphoid organs to the development of psoriasis-like skin inflammation in both these models. Gating strategies of innate immune cells from spleen and draining lymph nodes are shown in Supplementary Figure 1.

### Contribution of γδ T cells to the disease development

**Figure 5:**
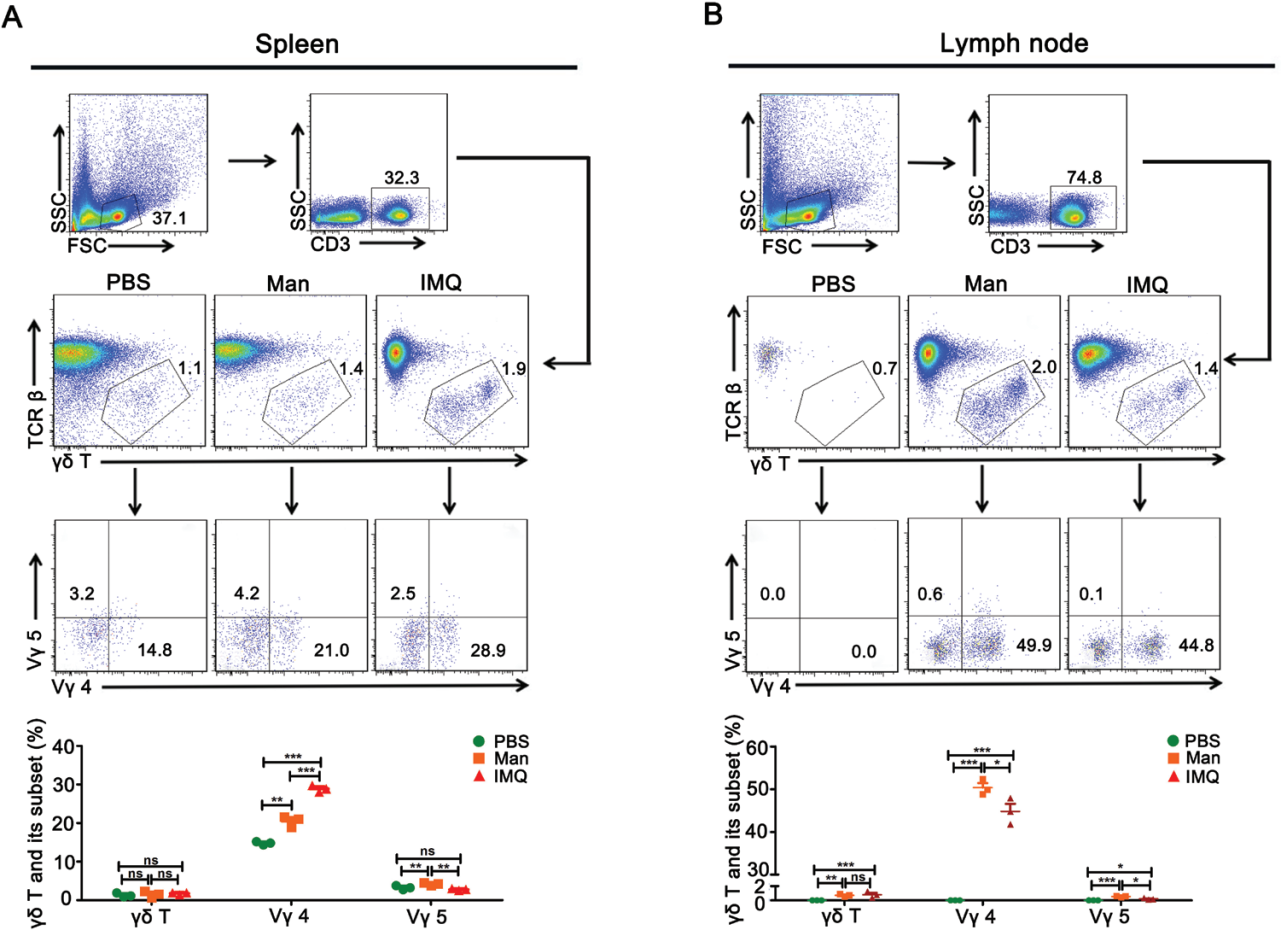
Percentage of γδ T cells and their subsets in the spleen and lymph nodes after mannan or IMQ application. (**A** and **B**) Representative pictures and gating strategies showing γδ T cells as well as their subsets (Vγ4 and Vγ5) in MISI and IISI at day 5 in the spleen and draining lymph nodes (*n* = 6/group). Man, mannan; IMQ, imiquimod. Statistical analyses were performed using an unpaired *t* test and *n*, indicates number of mice used in each group. The data represent mean ± SEM. ns, not significant; *P < 0.05; **P < 0.01; ***P < 0.001.

An increase in the level of IL-17A^+^ γδ T cells in the lymph nodes facilitate their migration to the skin where these cells persist as memory-like cells in IISI [16]. In this study, we compared the expression of γδ T cells and its subsets (Vγ4 and Vγ5) in the spleen and draining lymph nodes between mannan and IMQ applied mice. Negligible difference was observed in the total percentage of γδ T cells and Vγ5 cells in the spleen between MISI and control group, while a significantly increased expression of Vγ4 T cells seems to be dependent on the tested lymphoid organs between these two models (Figure 5A). Interestingly, a significant increase in the total γδ T cells was found in the draining lymph nodes after mannan or IMQ stimulation, which might be mainly due to the observed increase in the level of Vγ4 cells. However, both the Vγ4 and Vγ5 cells were found to be increased in the lymph nodes after exposure to mannan or IMQ (Figure 5B).

### Expression of pro-inflammatory cytokines and receptors

**Figure 6:**
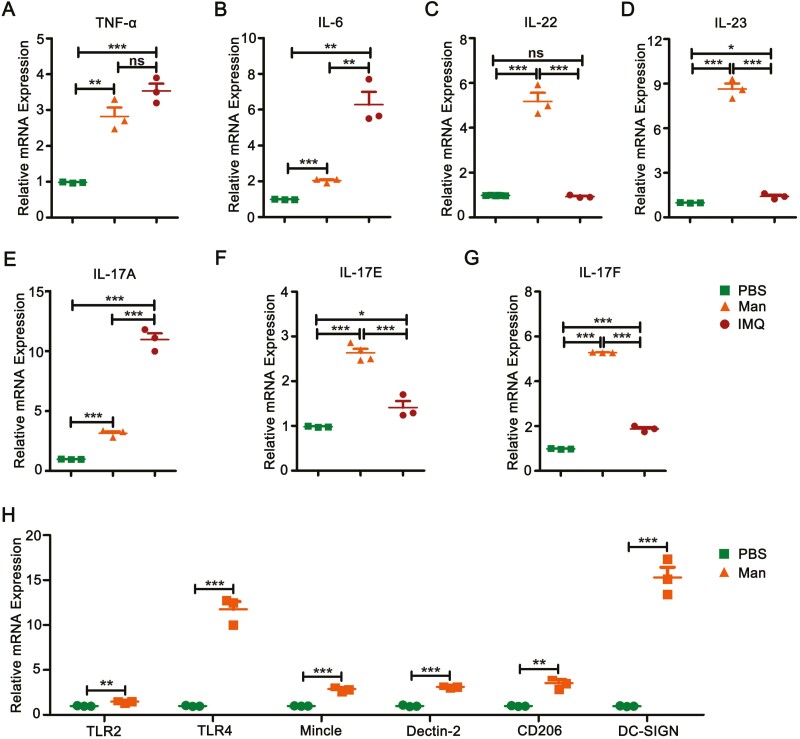
Expression of pro-inflammatory cytokines. (**A–C**) The qPCR results showing the expression of pro-inflammatory cytokines (IL-6. TNF-α , and IL-22) in MISI and IISI. (**D–G**) Expression of IL-23/IL-17 pro-inflammatory axis cytokines in MISI and IISI in the diseased skin (*n* = 6/group). (**H**) Relative mRNA expression of C type lectin receptors (TLR2, TLR4, Mincle, Dectin-2, CD206, and DC-SIGN) in MISI (*n* = 3/group). Man, mannan; IMQ, imiquimod. Statistical analyses were performed using an unpaired *t* test and *n*, indicates number of mice used in each group. The data represent mean ± SEM. ns, not significant; *P < 0.05; **P < 0.01. ***P < 0.001.

Psoriasis skin samples were harvested at the peak of psoriasis (day 5 for MISI, day 6 for IISI) and used to analyze the expression of genes of pro-inflammatory cytokines. IL-6 and TNF-α, the pro-inflammatory cytokines secreted by CD11c^+^ DCs and macrophages [17] were found to be significantly increased in both these models (Figure 6A-B). Acanthosis related cytokine IL-22 has significantly increased in MISI but not IISI (Figure 6C). However, an increased expression of IL-23/IL-17 inflammatory axis cytokines was found in both MISI and IISI (Figure 6D-G). Since specificity of IMQ to TLR7/8 has been well established [18], next, we investigated the expression of mannan binding receptors [19] in the skin samples after exposure to mannan. As expected, expression of all the mannan binding receptors (TLR2, TLR4, Mincle, Dectin-2, CD206, and DC-SIGN), were found to be higher in MISI, with a most prominent increase in the expression of TLR4 and DC-SIGN (Figure 6H).

### Dexamethasone decreased mannan and IMQ induce psoriasis

**Figure 7:**
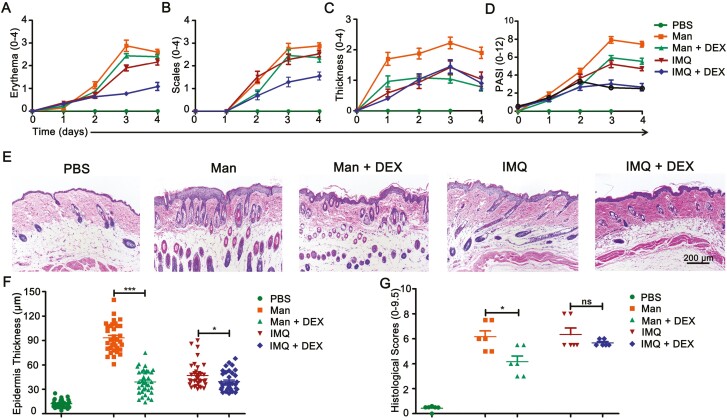
Treatment with dexamethasone. (**A–D**) Comparison of redness, scales, skin thickness, and total PASI between PBS, Man, Man + DEX, IMQ, IMQ + DEX groups (*n* = 10/group). (**E**) Representative pictures of H&E staining from psoriatic skin in MISI and IISI groups after dexamethasone treatment (*n* = 5/group). Scale bar: 200 μm. (**F**) Epidermal thickness measurements (*n* = 6/group) and (**G**) pathological scores (Baker’s scores) of histological sections (*n* = 6/group). Man, mannan; IMQ, imiquimod; DEX, dexamethasone. Statistical analyses were performed using an unpaired *t* test and *n*, indicates number of mice used in each group. The data represent mean ± SEM. ns, not significant; *P < 0.05; ***P < 0.001.

As a next step, we investigated how the mannan compared to IMQ-induced skin inflammation behaves after treatment with dexamethasone. A decrease in erythema (Figure 7A) and scales (Figure 7B) in IISI but not MISI was observed after dexamethasone treatment, while a significant decrease in skin thickness was observed in MISI (Figure 7C). Although dexamethasone treatment has significantly decreased psoriasis scores (Figure 7D) and epidermal thickness (Figure 7E-F) in both these models, histological scores were significantly decreased only in mannan but not IMQ-induced skin inflammation (Figure 7G).

## Discussion

Here, we described the development and characterization of a novel mannan-induced psoriasis mouse model in inbred mouse strains, which is similar to plaque psoriasis observed in patients. Redness covered with white scales accompanied with skin thickness in the lesional area were more obvious after mannan exposure. This model is easy to induce, robust, reproducible, and more economical for testing various potential drug candidates within a short duration of time. Most importantly, unlike IMQ-induced model, mannan exposure did not result in severe weight loss and, the disease relapse was more severe and robust, which could be exploited for developing a chronic psoriasis mouse model mimicking the disease development in patients more closely. Proliferation of keratinocytes and innate immune cells, infiltration of T lymphocytes in the skin, and an increase in the expression pro-inflammatory cytokines observed after epicutaneous mannan application, a natural route of antigen exposure in the skin, could have contributed to the observed skin inflammation. Moreover, dexamethasone treatment has significantly reduced PASI in MISI suggests usefulness of mannan-induced model in the development of new drugs and/or testing new combinations of available drugs to treat psoriasis patients.

Psoriasis is a chronic autoimmune disease having a strong genetic connection and certain known environmental triggers (smoking, emotional stress, and sex hormones), and it is mainly divided into five types: plaque, guttate, articular, erythrodermic, and pustular psoriasis. Among them, plaque psoriasis is the most common type and constitute up to 90% of the affected patient population [20]. It is characterized by a well-defined erythema covered with silvery white scales having an increase in the skin thickness. Histopathological analysis has shown an intense proliferation of keratinocytes in the epidermis leading to hyperkeratosis, parakeratosis, and vasodilation as well as infiltration of immune cells in the dermis [21]. Similarly, erythema, scales and thickness of lesional skin after mannan exposure were observed, in addition to an increase in the keratinocyte proliferation and immune cell infiltrations in the skin. Inflammatory molecules such as TNF-α, IL-6, CXCL10, and CCL20 secreted by keratinocytes can activate and recruit neutrophils, macrophages and dendritic cells to the lesional skin [22]. These immune cells in turn secrete IL-22, IL-23, and IL-17 family of cytokines, which results in the proliferation of keratinocytes and forms a positive feedback regulation. Neutrophils, macrophages and dendritic cells present in the skin and secondary lymphoid organs contribute to the inflammation in both the psoriasis models but the percentage of individual cell populations do differ between the models at the peak of disease induction.

Recently, the γδ T cells, which are in between innate and adaptive immunity, have received more attention in psoriasis. Exposure to mannan has significantly increased the level of γδ T cells in both the lymph nodes and spleen, especially Vγ4 subset. Recent studies showed that naive γδ T cells could develop into immune memory cells after encountering the same antigen again, and Vγ4 and Vγ5 T cells are the main memory phenotypes within the γδ T cell population in the mouse skin [23]. Interestingly, dermal Vγ4 T cells were reported to be enhanced in IMQ re-challenged mice [24]. Therefore, stronger psoriasis symptoms observed during disease relapse after mannan application suggest a major contribution from Vγ4 T cells during this disease phase. At the same time, the importance of IL-17A producing Vγ4^+^ γδ T cells in the skin was well emphasized in the IMQ model [24]. Similarly, studies have been done to understand the critical contribution of these cells present in the skin exposed to mannan (Khmaladze et al., unpublished work). However, specific experiments with multiple mannan exposure at different time points with γδ T cell KO mice as controls are needed to prove this notion.

CD4^+^ αβ T cells, CD8^+^ αβ T cells, NKT cells, and γδ T cells, as well as non-T cells like macrophages and neutrophils produce IL-17. However, γδ T cells are often the major source of this cytokine [25, 26], especially during the early stages of a disease. It was earlier shown that IL-23 has predominantly stimulated dermal γδ T cells, which expressed the IL-23R constitutively, to produce IL-17 that led to the progression of IISI in mice. Moreover, in psoriasis patients, γδ T cells were also greatly increased in the affected skin and produced large amounts of IL-17 [27]. IL-23/IL-17 axis was earlier shown to be important in inducing mannan [14] and IMQ [28, 29] induced psoriasis in mice by neutralizing these cytokines. IL-23 is critical for differentiation, survival, and expansion of IL-17 producing (Th17 and γδT) cells [30, 31]. Mice lacking IL-17RA or IL-23p19 [7] had significantly reduced level of psoriasis upon exposure to the Aldara cream, especially IL17RA in keratinocytes seems to have an important contribution to psoriasis development [32]. The rhIL23R-CHR/Fc fusion protein was shown to inhibit immune-mediated inflammatory responses and acted as a natural antagonist in IISI [33].

TNF-α and IL-6 secreted by hyper-proliferating keratinocytes contribute to the recruitment of various immune cells, such as neutrophils and CD11c^+^ DCs [1]. IL-22, one of IL-17 family cytokines, secreted by Th17 cells, Th1 cells as well as γδ T cells, has a crucial function in the development of dermal inflammation and epidermal acanthosis [34]. Moreover, IL-23 secreted by dendritic cells and macrophages stimulate γδ T cells to secrete IL-17A, IL-17F, and IL-22 in psoriasis [27, 35]. We observed an overexpression of TNF-α, IL-6, IL-22, IL-23, and IL-17 family cytokines (IL-17A, IL-17E, and IL-17F) in mannan-induced psoriasis-skin inflammation, suggesting their contribution to the inflammatory processes in the affected skin, similar to the reported role of these cytokines in IISI [7]. However, time kinetics and global gene expression analysis are further needed to better understand the expressions of the cytokine genes in psoriasis.

The mechanisms behind mannan-induced psoriasis and psoriasis arthritis were well studied. After injection of mannan, it rapidly spreads to immune system, skin, and joints within 6 h [36] and increased the psoriasis severity by promoting the differentiation of pDCs via the STAT3-IRF8 pathway [37]. Apart from macrophage mannose receptor involved in the regulation of psoriasis [38], mannan was shown to promote keratinocytes to secrete CXCL1 and enhance the infiltration of neutrophils during the disease [39]. Furthermore, disturbances in the antioxidant system, and an increased production of ROS, contributed to psoriasis pathogenesis as well [40, 41] especially the mitochondrial ROS [42]. Mannan-induced macrophages produced TNF-α, which stimulated local γδ T cells secreting IL-17A and the neutralization of IL-17A and depletion of granulocytes blocked the disease symptoms [14]. Similarly, deficiency of Qa2 due to natural loss of function mutations increased IL-17 producing γδ T cells and group 3 ILCs in the draining lymph nodes of mice with mannan-induced psoriasis [43]. In this context, mannan-induced Nos2 in macrophages was shown to have a prominent role in enhancing IL-17 promoted psoriatic arthritis by innate immune cells [44]. Recently, we have also shown the pathogenic role of estrogen in psoriasis after epicutaneously applying the mannan in mice [45]. Therefore, in this study, we focused on the differences in the clinical symptoms, histological features, and drug treatment between MISI and IISI models to highlight the advantages of using mannan-induced psoriasis model for further studies. Exposure of mannan via subcutaneous or intraperitoneal injection routes may not have any major differences in terms of underlying systemic inflammatory mechanisms, though the application on the skin can be considered as more natural and the local immune and inflammatory mechanisms do vary depending on the route of antigen delivery.

Antigens binding to different types of pattern recognition receptors (PRRs), such as TLRs, CTRs, NLRs, and MR, could result in a cascade of immune responses during psoriasis development [46]. Effects of IMQ are mostly mediated by the activation of TLR7 and TLR8 expressed on the immune cells [47], whereas mannan was shown to activate mannose receptor (CD206) on macrophages in psoriasis, psoriatic arthritis, and rheumatoid arthritis-like disease models [38]. Apart from CD206, mannan binds to various other receptors as well [19]. In this study, we found a higher level expression of TLR2, TLR4, CD206, Dectin-2, Mincle, and DC-SIGN after mannan exposure. This observation suggests two potential advantages in using mannan for induction of psoriasis: (i) Mannan, unlike IMQ, by activating various receptors present on the immune cells could possibly mimic natural environmental factors affecting the skin more closely that are responsible for psoriasis development; (ii) MISI model could potentially be used to explore the relative contribution of different PRRs in the development and severity of psoriasis as well for testing different therapeutic strategies specifically targeting individual PRRs.

It is plausible that the topical application of mannan plus an adjuvant in the skin activates C type lectin-like receptors and toll-like receptors (Figure 6H), present on the keratinocytes and dendritic cells, which may trigger conditions that promote IL-23 production, as demonstrated in the case of microbes present on the skin [48, 49]. IL-23 is not only secreted by dendritic cells but also produced by keratinocytes [50] and other activated antigen-presenting cells like Langerhans cells and macrophages as well that are present in the skin [51]. Whereas, after IMQ application, IL-23 was reported to be exclusively produced by Langerin^neg^ DCs *in vivo* [52], and needed for the development of IISI by induction of IL-17A producing γδ T cells [4]. Earlier, IL-23 secreted by these cells was shown to stimulate γδ T cells to secrete IL-17A, IL-17F, and IL-22 in psoriasis [27, 35]. In addition, some of the γδ T cells were shown to express IL-23R and the transcription factor RORγt and produce IL-17, IL-21, and IL-22 in response to IL-1β and IL-23 [30].

Unlike IMQ that requires 5 or 7 days of application, mannan needs to be applied only for 3 days for disease induction, thereby reducing the cost of screening several potential drug candidates. In addition, MISI does not cause severe weight loss or death of mice and the disease induced imitate plaque psoriasis phenotype observed in patients. Moreover, a stronger treatment response to dexamethasone suggests usefulness of this model for further drug development in the future. Despite inducing psoriasis-like inflammation, certain differences and similarities were observed in the immune responses induced by mannan and the commonly used IMQ. However, mannan-induced psoriasis model is relatively more simple, economical and less harmful to mice with the possibility for developing a chronic psoriasis model for future studies exploring disease mechanisms, genetics, and evaluation of potential drugs.

## Supplementary data

Supplementary data is available at *Clinical and Experimental Immunology* online.

**Supplementary Figure 1**. Gating strategies for innate immune cells (dendritic cells, macrophages and neutrophils) from the spleen and lymph nodes.

uxad004_suppl_Supplementary_Figure_Legend

uxad004_suppl_Supplementary_Figure_S1

## Data Availability

Data are available for further analysis upon reasonable request.

## Abbreviations

CFA: complete Freund’s adjuvant
DCs: dendritic cells
IFA: incomplete Freund’s adjuvant
IISI: imiquimod-induced skin inflammation
IMQ: imiquimod
MISI: mannan-induced skin inflammation
PASI: psoriasis area and severity index

## Permission to reproduce (for relevant content)

After obtaining permission from publishers.
