# Supplementary material for: Comparative studies on mannan and imiquimod induced experimental plaque psoriasis inflammation in inbred mice
Source: Clin Exp Immunol. 2023 Jan 16;211(3):288–300. doi: 10.1093/cei/uxad004 (PMC10038325; doi:10.1093/cei/uxad004)
Supplement: uxad004_suppl_Supplementary_Figure_Legend [file uxad004_suppl_supplementary_figure_legend.docx]

**Supplementary figure 1**: Gating strategies for innate immune cells (dendritic cells, macrophages and neutrophils) from the spleen and lymph nodes.
