# Supplementary figures and images for: Comparative studies on mannan and imiquimod induced experimental plaque psoriasis inflammation in inbred mice
Source: Clin Exp Immunol. 2023 Jan 16;211(3):288–300. doi: 10.1093/cei/uxad004 (PMC10038325; doi:10.1093/cei/uxad004)

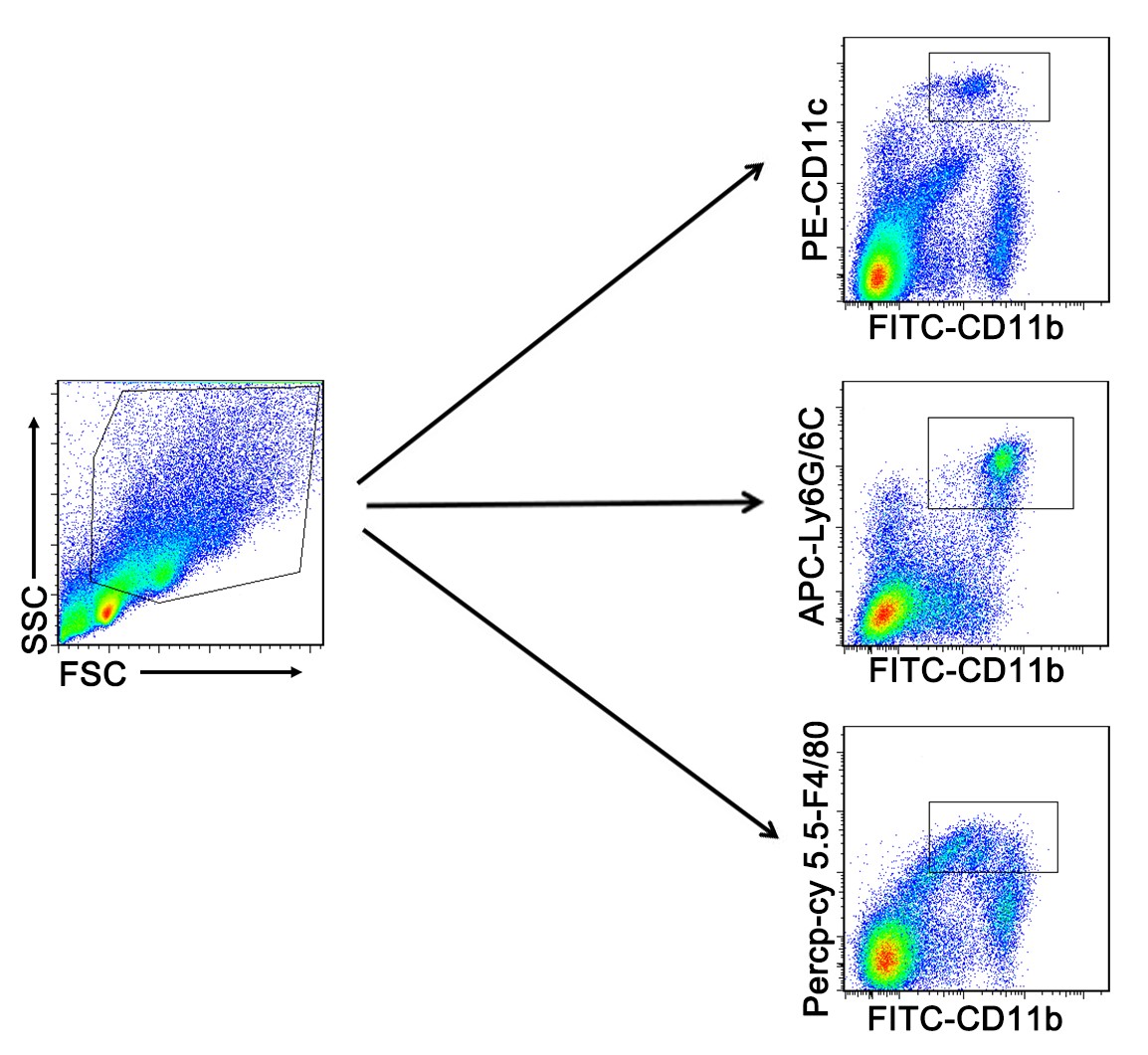

Supplement: uxad004_suppl_Supplementary_Figure_S1 [file uxad004_suppl_supplementary_figure_s1.jpeg]
